# Presence of Potential Toxin-Producing Cyanobacteria in an Oligo-Mesotrophic Lake in Baltic Lake District, Germany: An Ecological, Genetic and Toxicological Survey

**DOI:** 10.3390/toxins6102912

**Published:** 2014-09-29

**Authors:** Pawan K. Dadheech, Géza B. Selmeczy, Gábor Vasas, Judit Padisák, Wolfgang Arp, Kálmán Tapolczai, Peter Casper, Lothar Krienitz

**Affiliations:** 1 Leibniz-Institute of Freshwater Ecology and Inland Fisheries, Alte Fischerhütte 2, Stechlin D-16775, Germany; E-Mails: pdadheech@curaj.ac.in (P.K.D.); pc@igb-berlin.de (P.C.); 2 Department of Microbiology, Central University of Rajasthan, Bandarsindri-305801 (Ajmer), India; 3 Department of Limnology, University of Pannonia, Egyetem utca 10, Veszprém H-8200, Hungary; E-Mails: selmeczy.geza@gmail.com (G.B.S.); padisak@almos.uni-pannon.hu (J.P.); 4 Department of Botany, University of Debrecen, Egyetem tér 1, Debrecen H-4032, Hungary; E-Mail: vasas.gabor@science.unideb.hu; 5 MTA-PE Limnoecology Research Group, Egyetem utca 10, Veszprém H-8200, Hungary; 6 LimPlan, Gewässer- und Landschaftsökologie, Otawistr. 19, Berlin D-13351, Germany; E-Mail: w.arp@limplan.de; 7 INRA, UMR CARRTEL, 75 avenue de Corzent, BP 511, Thonon-les-Bains Cedex F-74203, France; E-Mail: kalman.tapolczai@thonon.inra.fr

**Keywords:** cyanotoxins, microcystin, Lake Stechlin, *Aphanizomenon*, *Dolichospermum*, *Microcystis*, *Planktothrix*

## Abstract

Massive developments of potentially toxic cyanobacteria in Lake Stechlin, an oligo-mesotrophic lake in the Baltic Lake District of Germany raised concerns about toxic contamination of these important ecosystems. Field samples in the phase of mass developments of cyanobacteria were used for genetic and toxicological analyses. Microcystins and microcystin genes were detected in field samples of the lake for the first time. However, the toxins were not produced by the dominant taxa (*Dolichospermum circinale* and *Aphanizomenon flos-aquae*) but by taxa, which were present only in low biomass in the samples (*Microcystis* cf. *aeruginosa* and *Planktothrix rubescens*). The phytoplankton successions during the study period revealed an increase of cyanobacterial populations. The findings contribute to the changes that have been investigated in Lake Stechlin since the mid-1990s. The possible reasons behind these developments may be climate change, special weather conditions and an increased nutrient pool.

## 1. Introduction

The Baltic Lake District in northeastern Germany hosts a multitude of lakes formed during the last glacial period (~12,000 years before) [1]. Some of the lakes are pristine and belong to the highest classes of water quality according to the European Water Framework Directive (WFD). Lake Stechlin represents a highly valuable ecosystem. The lake belongs to the type 13 of WFD that collects stratified lowland lakes with small catchment area with high content of calcite [2].

The investigation of phytoplankton started in 1959, the year the Limnological Laboratory Stechlin was founded. Since then, a number of investigations in relation to the different aspects of phytoplankton were performed: species composition, temporal and spatial distribution of the dominant species, seasonal periodicity, cell number and biomass calculation. These studies were conducted using different sampling frequencies. The first results were published by Busse [3] and Küchler [4,5]. Later, Casper [1] synthesised the periods of 1959–1962, 1963–1964, 1969–1972, and 1976–1978. Afterwards, many articles appeared on the phytoplankton of Lake Stechlin. One of the newest articles synthesises the period of 1994–2008, based on biweekly sampling of phytoplankton [6]. These regular detailed studies allow researchers to follow long-term trends. Mass developments of cyanobacteria were found only since the early 21st century. *Aphanizomenon*, *Dolichospermum* and *Planktothrix* dominated, while *Microcystis* was not found in masses.

In freshwaters, cyanobacteria are the main producers of potentially toxic blooms. Such mass developments can negatively influence ecosystems, as well as human and animal health, in addition to restricting the water’s multipurpose use (e.g., for drinking water, fishing, recreation) [7,8,9,10,11]. The increasing frequency of toxic cyanobacterial blooms in freshwater ecosystems is related to eutrophication [12]. The potential of toxin production differs between the dominating cyanobacteria genera. Mainly *Dolichospermum* (former *Anabaena*), *Microcystis* and *Planktothrix* are known toxin producers [13,14]. When mass developments of these genera occur, surveys for toxins are recommended in order to avoid impact on humans or animals [15]. Among all cyanotoxins found in the environment, microcystin (MCYST) and nodularin are the most common hepatotoxins that are produced by a wide range of cyanobacteria [16,17].

Recently, during a survey in the northeastern lakes of Germany, Ballot *et al.* [18] confirmed the occurrence of cyanotoxins (saxitoxin, anatoxin-a and cylindrospermopsin) and genotypes of putative saxitoxin-producing *Aphanizomenon flos-aquae* and *Anabaena planktonica* in Lake Stechlin. Earlier, microcystin and putative microcystin-producing species (*Planktothrix rubescens* HUB151) were detected in Lake Stechlin [19]. Apart from these studies, no additional sources of microcystin production have been identified in Lake Stechlin.

Over the past few years, a rapid invasion by nostocalean cyanobacteria has occurred, and a number of *Dolichospermum* species, together with *Aphanizomenon flos-aquae* and oscillatorian *Planktothrix agardhii*, have become established in the lake [6]. The abundance of potentially toxic cyanobacteria has increased during the last decade [6,20].

Lake Stechlin is an ecological model water for studying the influence of climate change on lakes. The Leibniz-Institute of Freshwater Ecology and Inland Fisheries, with a 55-year history of studying the lake, installed a large experimental platform, the LakeLab [21]. Furthermore, Lake Stechlin is a well-known tourist attraction in northeastern Germany, and during the summer, it is a popular bathing site. Hence, increasing our knowledge on cyanobacterial mass developments and the occurrence of cyanotoxins in this lake is crucial, as this lake is a model for other nutrient-poor lakes.

Considering the increasing frequency and intensity of cyanobacterial blooms in temperate aquatic ecosystems due to climate change and the scarcity of reports on potential toxins, particularly MCYST, produced by cyanobacteria in lakes of the Baltic Lake District of Germany, this study aimed at detecting cyanobacterial strains that have the genetic potential to produce microcystin and other cyanotoxins, as well as to conduct an analysis of MCYST from cyanobacterial water blooms in Lake Stechlin. In this study, we not only investigated mass-developing cyanobacteria, but also those with minor abundance for toxicological and molecular genetic investigations.

## 2. Results

### 2.1. Phytoplankton Study

**Figure 1:**
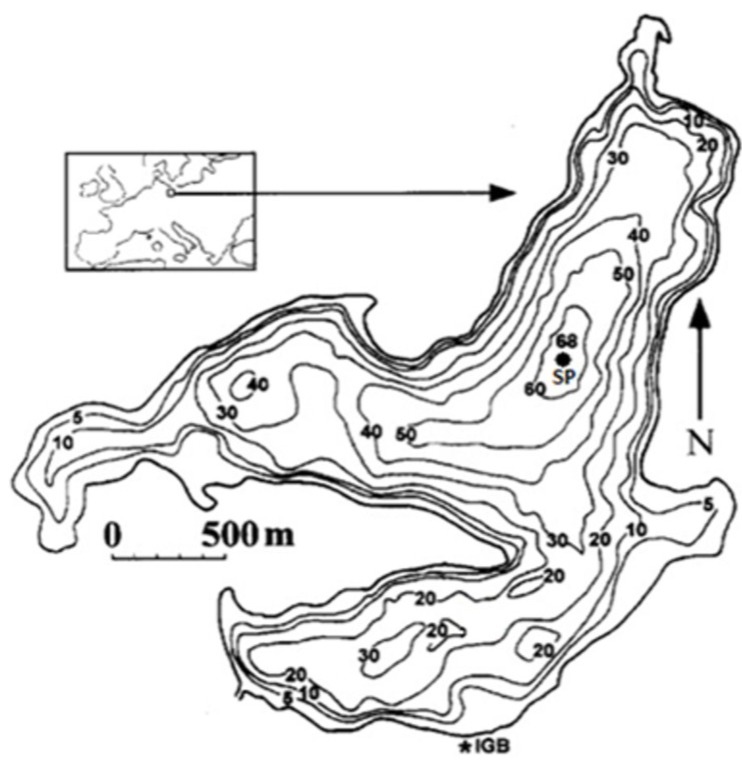
Bathymetric map of Lake Stechlin (SP = sampling point of pelagic phytoplankton; IGB = location of the Institute and sampling point of littoral samples).

Lake Stechlin is a glacial originated, deep, oligo-mesotrophic lake located in the Baltic Lake District (53°10′N, 13°02′E; 59.9 m above sea level, Figure 1). The surface temperature increased over the last 50 years by 0.37 °C per decade to an annual mean of 11.3 ± 0.5 °C [22], with effects on stratification, heat exchange, biological activities *etc.* For example, since 2000, the surface temperature was higher than 20 °C on at least one sampling day of the year; in 2003, 2006, and 2009, this period extended to five days (data not shown).

The P- and N-concentrations on the two sampling dates (Table 1) were typical for oligo- to mesotrophic lakes. In November, at the end of the stratification period, an accumulation of the nutrients in the hypolimnion was found (e.g., TP 0.113 mg L^−1^ and SRP 0.104 mg L^−1^). A decrease in oxygen saturation from 103.8%–49.6% was observed in the deep layers.

**Figure 2:**
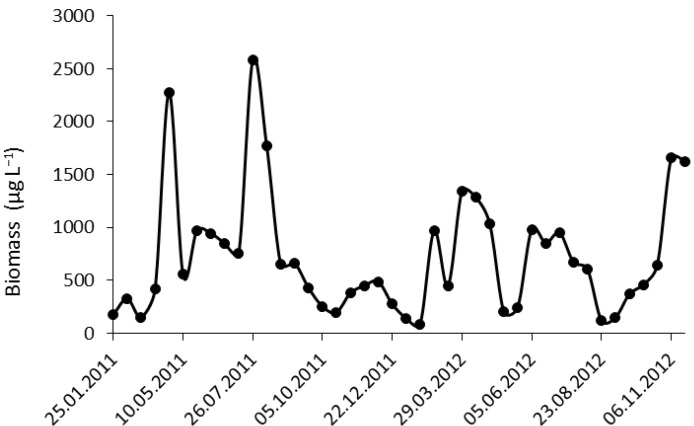
Phytoplankton biomass in Lake Stechlin during 2011 and 2012.

**Figure 3:**
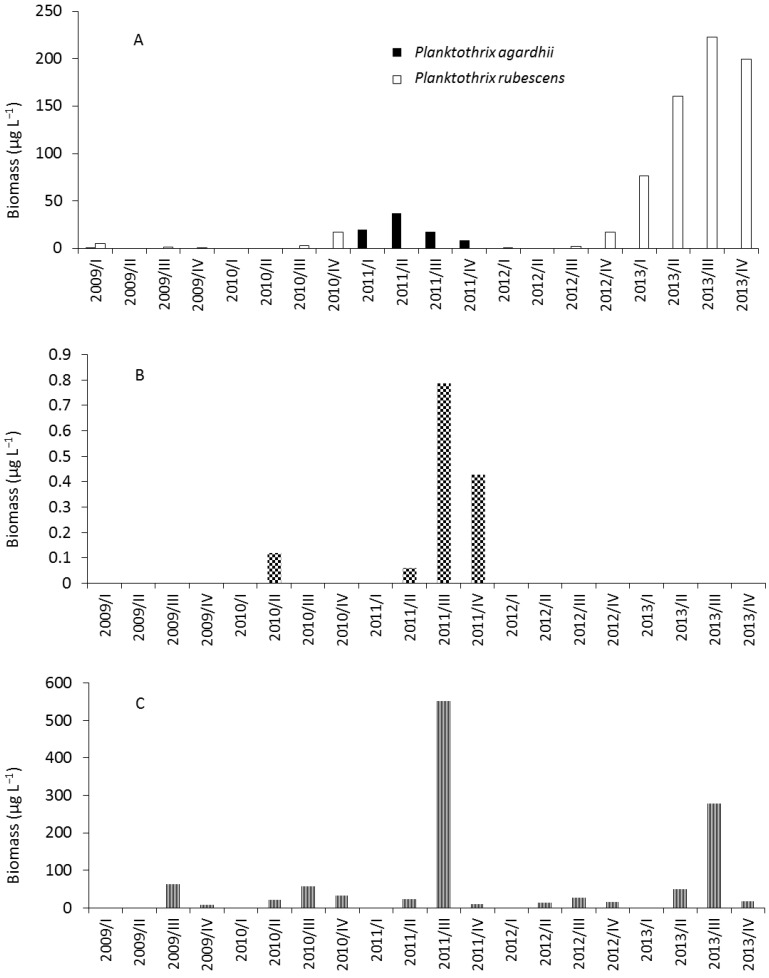
Average biomass of *Planktothrix rubescens*, *Planktothrix agardhii* (**A**); *Microcystis* cf. *aeruginosa* (**B**); and *Dolichospermum* spp. (**C**) between 2009 and 2013 in Lake Stechlin. I, II, III, and IV stand for quarters of years.

Two peaks of phytoplankton biomass were found during 2011 (Figure 2). The first peak was in April and was dominated by centric diatoms, such as *Stephanodiscus neoastraea* (maximum: 1779 µg L^−1^). The second peak occurred in July and was dominated by *Dolichospermum* species, mainly *Dolichospermum circinale* (maximum: 1198 µg L^−1^), and additionally by the dinoflagellate *Ceratium hirundinella* (510 µg L^−1^). Notably, *Microcystis* cf. *aeruginosa* existed in Lake Stechlin above the observation limit, mainly during the second half of 2011 (Figure 3).

**Table 1:** P- and N-concentrations; physical properties in July 2011 and November 2012.

Sampling time	Water Layers	TP (mg·L^−1^)	SRP (mg·L^−1^)	TN (mg·L^−1^)	DIN (mg·L^−1^)	pH	Conductivity (µS·cm^−1^)	O_2_ (mg·L^−1^)	O_2_ (%)	Secchi depth (m)
July 2011	Epilimnion	0.011	0.002	0.376	0.039	8.70	285	10.87	122.4	5.7
	Hypolimnion	0.020	0.009	0.334	0.064	8.17	292	11.99	99.5	
Nov 2012	Epilimnion	0.010	0.003	0.393	0.024	7.92	282	10.52	91.3	5.5
	Hypolimnion	0.113	0.104	0.425	0.155	7.02	298	4.27	34.0	

Epilimnion: 0–5 m (July, 2011), 0–10 m (November, 2012); Hypolimnion: 10–65 m (July 2011), 15–65 m (November 2012).

**Figure 4:**
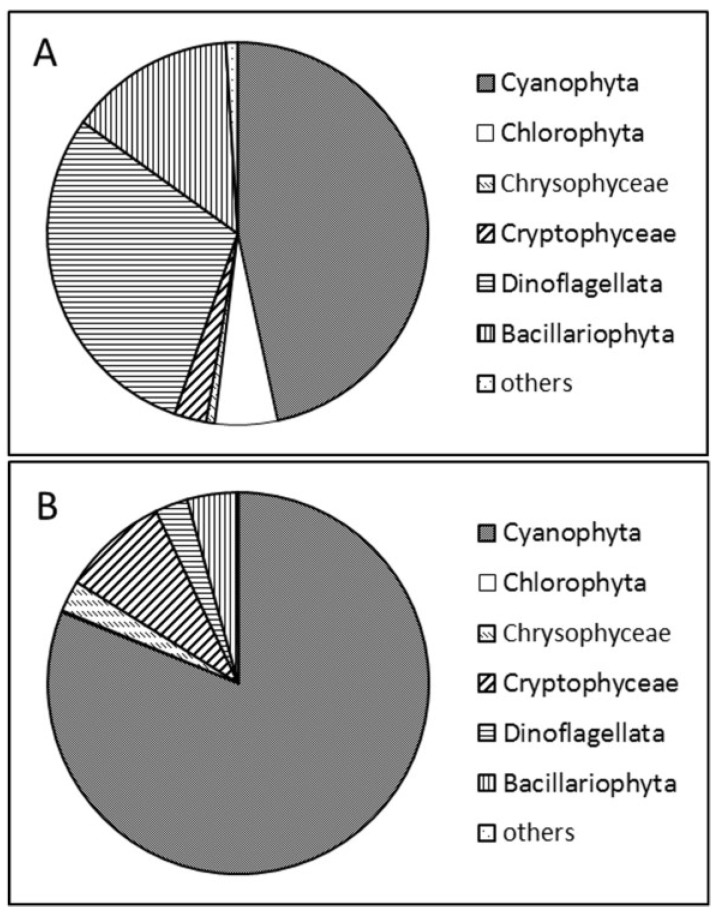
Ratio of pelagic phytoplankton communities in July 2011 (**A**) and November 2012 (**B**).

In 2012, three peaks were observed. Large centric diatoms, like in 2011, established the first peak of the year. The second peak was lower and the phytoplankton assemblage was more diverse than in 2011. *Asterionella formosa* was the dominant species during early June, and later cryptophytes and dinoflagellates were observed with high biomass. The third peak was a very unusual event regarding the general phytoplankton succession of Lake Stechlin, since the phytoplankton assemblage was dominated by *Aphanizomenon flos-aquae* with >80% contribution to total biomass. Besides, *Planktothrix rubescens* occurred in increasing amounts (60 µg L^−1^) (Figure 3). At both field samplings of the lake, which took place in July 2011 and November 2012, cyanobacteria dominated the pelagic phytoplankton community (Figure 4).

### 2.2. Molecular Analyses

**Figure 5:**
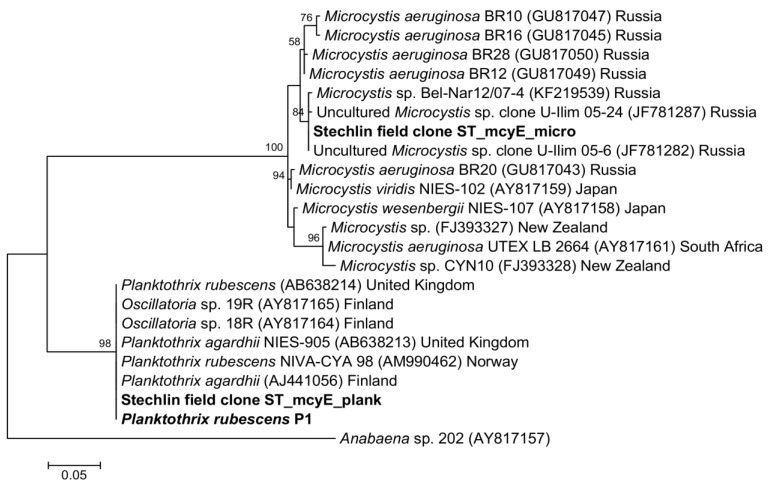
Maximum likelihood tree based on partial nucleotide sequences of the *mcy*E gene showing the phylogenetic relationship among sequences from field samples and isolates from Lake Stechlin (bold), with reference sequences obtained through BLASTN analysis. Bootstrap values >50 were included.

The sequences of the AMT domain for the *mcy*E gene obtained in this study showed the highest similarity to *Microcystis* and *Planktothrix*, respectively, in the BLASTN analysis. We gained 386 bp sequences from amplified products of the *mcy*E gene. These sequences, together with closely-related retrieved sequences from GenBank were aligned and the inferred phylogeny of the *mcy*E gene, using 351 positions, was determined. The phylogenetic tree depicted a clear bifurcated topology with a clade, including sequences mostly from *Microcystis*, while the other clade was dominated by sequences of *Planktothrix* (Figure 5). The *Microcystis* clade was supported by a 100% bootstrap value, and likewise, the *Planktothrix* clade was supported by a bootstrap value of 98%.

The sequence of the Stechlin field clone ST2011_mcyE_micro and of the isolated *Microcystis* strains (M1 and M2) were integrated into the clade-comprising sequences of *Microcystis* strains from different countries. The *mcy*E sequences attained from Lake Stechlin were clustered with sequences from Russia. The *mcy*E sequences of clones obtained from the field sample in 2012 and a strain of *P*. *rubescens* (P1) were included in a clade with sequences of different species of *Planktothrix* and other members of Oscillatoriales from European origin. A sequence of the *mcy*E gene fragment amplified with HEPF/HEPR primers belonging to *Planktothrix* and *Microcystis* was not available in GenBank for comparison. Isolated strains of *Dolichospermum circinale* (A1, A5 & A7), *Aphanizomenon* *flos-aquae* (AP1) were also tested with HEPF/HEPR primers, but without any amplification for the *mcy*E gene. PCR analyses of the field samples and isolates were also performed using specific primer pairs for other cyanotoxins (saxitoxin and anatoxin-a), but we did not get any amplification.

**Figure 6:**
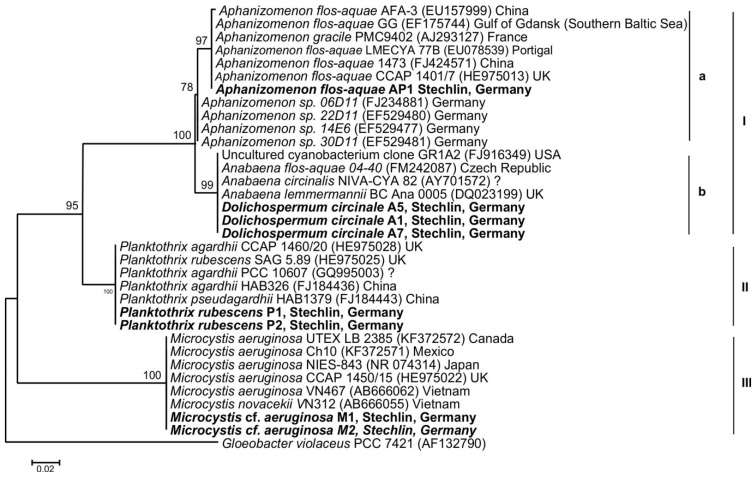
Maximum likelihood tree based on partial 16S rRNA gene (V3 and V4 regions) sequences showing the phylogenetic relationship among the sequences of the cyanobacterial strains from Lake Stechlin (bold), with reference sequences obtained through BLASTN analysis. Bootstrap values >50 were included. I, II, III stand for main clades and a, b stand for sub-clades.

A partial sequence of the 16S rRNA gene (V3 and V4 regions) were used to evaluate the phylogenetic position of *Dolichospermum circinale*, *Aphanizomenon flos-aquae*, *Microcystis* cf. *aeruginosa* and *Planktothrix rubescens* strains isolated from Lake Stechlin. We could get 396 bp sequences of 16S rRNA gene fragment from all studied strains and the same number of positions used for phylogenetic analysis. The phylogenetic tree showed three well-differentiated clades (Figure 6). Clade I was divided into two sub-clades (Ia and Ib), comprised of different strains of *Aphanizomenon* and *Anabaena* supported by 100% bootstrap, respectively. The Lake Stechlin sequence of *Aphanizomenon flos-aquae* AP1 fell in the sub-clade (Ia) containing strains that were mostly not originated from Germany. This sub-clade was supported by a high bootstrap value (97%). Sequences of German strains formed a separate sub-clade (Ib). Sequences of *Dolichospermum* strains (A1, A5 and A7) corresponded to *Anabaena lemmermanni* with 100% similarity and were included in the sub-clade (Ia) in which various *Anabaena* species were present. This sub-clade had support of 99% bootstrap. There were a few sequences of 16S rRNA gene of the genus *Anabaena* belonging to species *A*. *bergii* (EF529483, EF 529484) that were available in GenBank, but they created a distinct clade in the tree (data not shown). Thus, these sequences were not included in the presented phylogenetic tree. Another two tight clades (II and III) were formed and clade II had sequences related to *Planktothrix*, whereas clade III possessed sequences of *Microcystis*, originating from different countries, including our strains (Figure 6). Sequences of the V3 and V4 regions belonging to *Planktothrix* and *Microcystis*, which originated from Germany, were not available for comparison by that date from GenBank.

### 2.3. Cyanotoxin Analyses

**Figure 7:**
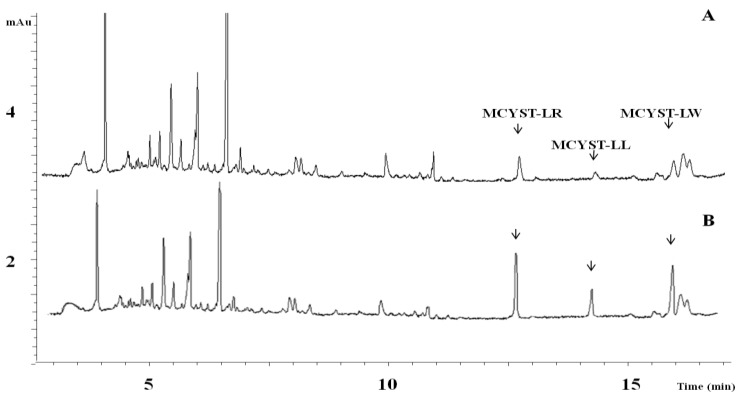
Micellar electrokinetic chromatographic (MEKC) separation of a plankton sample (July 2011) collected from Lake Stechlin (**A**) and the analysis of spiked sample (**B**) with 1 µg L^−1^ MCYST-LR, -LL, -LW (separation conditions: capillary: 64.5 cm, 50 µm i.d., buffer electrolyte: 25 mM borate and 0–100 mM SDS, pH = 9.3, applied voltage: +25 kV, detection: UV absorption at 238 nm).

The highest MC content was measured in the field sample from July 2011, and 27.32 µg L^−1^ was calculated as the MC-LR equivalent. The low MC content that was measured in the field sample from November 2012, and 4.25 µg L^−1^ was calculated as the MC-LR equivalent. Chemical forms of microcystin were detected by MALDI-TOF analyses in environmental samples as MCYST-LL, -LR, -LW, and -RR (Table 2). [Dha^7^]MCYST-RR variant was detected from a *P*. *rubescens* strain isolated from Lake Stechlin. A typical electrogram of a field sample from July 2011 showed different migrations of MCYST variants (Figure 7).

**Table 2:** Microcystin variants and contents detected in field samples and cyanobacterial strains from Lake Stechlin.

Sample/Strain	Dominant Taxon in Field Sample	Other Taxa in Field Sample	Microcystin Content (µg L^−1^ LR Equivalent)	Microcystin Variants	Toxin Gene Detected
July 2011	*Dolichospermum circinale*	*Microcystis* cf. *aeruginosa*	27.32 µg L^−1^	MCYST-LL, -LR, -LW, -RR	*mcy*E *Microcystis*
November 2012	*Aphanizomenon flos-aquae*	*Planktothrix rubescens*	4.25 µg L^−1^	MCYST-LW, [Dha7]MCYST-RR	*mcy*E *Planktothrix*
*Dolichospermum circinale* A1			n.d.		n. d.
*Dolichospermum circinale* A5			n.d.		n. d.
*Dolichospermum circinale* A7			n.d.		n. d.
*Aphanizomenon flos-aquae* AP1			n.d.		n. d.
*Microcystis* cf. *aeruginosa* M1			n.d.		n. d.
*Microcystis* cf. *aeruginosa* M2			n.d.		n. d.
*Planktothrix rubescens* P1			n.d.	[Dha7]MCYST-RR	*mcy*E *Planktothrix*
*Planktothrix rubescens* P2			n.d.		n. d.

n.d.: not detected.

## 3. Discussion

In general, the annual phytoplankton biomass distribution showed either a unimodal pattern, with a peak in the spring, or a bimodal pattern with a lower biomass peak in the late summer followed by the one in the spring [23]. Year 2011 was exceptional; the summer peak was higher than was the spring peak. The fact that the cyanobacteria dominated the summer assemblage and could reach a higher biomass than the diatoms that dominated the spring assemblage indicates a shift from oligo-mesotrophic towards a eutrophic state [24]. Until 2005, the epilimnetic summer assemblage was dominated mainly by dinoflagellates and chroococcalean blue-greens. Between 2006 and 2008, the *Aphanizomenon flos-aquae* and *Dolichospermum* species appeared, and then increased gradually until becoming dominant in the summer assemblage [6]. In recent years, the highest phytoplankton biomass peak was regularly caused by these species. It is well-known that the physiological optima for the photosynthesis of many cyanobacteria species are above 20 °C [25,26]. Lake Stechlin underlies climate change with significantly higher surface water temperatures, measured since 1958 as 0.37 °C per decade [22]. The duration of periods with at least a 20 °C surface temperature at Lake Stechlin is increasing, which may open a new ecological niche and contribute to the increased biomass of the *Dolichospermum* species.

In most of the cases, the phytoplankton biomass reached a minimum level after the autumnal overturn, when the relative water column stability (RWCS) value is near to zero, which occurs around November–December [23]. In autumn 2012, an unusual event was observed in the phytoplankton succession. After a minimum level of the phytoplankton biomass occurred in September, it continued increasing until early November, with *Aphanizomenon flos-aquae* being the dominant member of the phytoplankton assemblage.

Invasive species can have a major impact on the community composition and ecosystem processes [27,28]. *A*. *flos-aquae* is a native species in German lakes with high trophic state [29], but recently also appeared in the oligo-mesotrophic Lake Stechlin. The species was observed first in 2001, and until 2006, its occurrence was sporadic. It has started to increase over the past five years, and in some samples, >80% of the total biomass was contributed by *A*. *flos-aquae*.

The annual biomass pattern of *Planktothrix rubescens* in autumn 2012 showed a similarly increasing trend; however, during winter, *P*. *rubescens* reached a higher biomass than did *A*. *flos-aquae*. The surface appearance of the cold-water stenotherm *P*. *rubescens* in November is a common phenomenon [30]. *P*. *rubescens* has been present in the flora since the earliest records on phytoplankton, but it is usually rare and reaches dominance only in exceptional years. This occurred in 1998, when *P*. *rubescens* was the dominant species during almost the entire year, thus the general pattern of phytoplankton succession changed [31]. Padisák *et al.* [6] mentioned *P*. *rubescens* as an ecosystem engineer species, but in the last years, *A*. *flos-aquae*, together with the *Dolichospermum* species, were able to drive the phytoplankton succession during the whole year, except during the spring. Thus, these species are playing a key role in the phytoplankton assemblage of the no-longer oligotrophic Lake Stechlin.

The mass development of *Dolichospermum circinale* and *Aphanizomenon flos-aquae* was recorded in the months of July 2011 and November 2012, respectively. Besides the dominant cyanobacterial taxa, other cyanobacteria (*Planktothrix*, *Microcystis*) were observed. Putative microcystin-producing taxa *Microcystis* sp. and *Planktothrix agardhii* were documented in the samples collected in July 2011. *P*. *rubescens* was perceived during mass development of *Aphanizomenon flos-aquae* in November 2012. A microscopic analysis is not sufficient to monitor toxin-producing potential in blooms. Thus, the use of molecular markers that may detect only the toxic populations, independently of their taxonomic identity, is extremely effective [7].

To find out putative cyanotoxin-producing taxa, environmental samples of both sampling months and isolates were tested for the presence of various toxin-producing genes. For this purpose, we used selective primers to detect genes for the synthesis of cyanotoxins (anatoxin-a and saxitoxin, microcystin). The sequencing of amplified products confirmed the presence of cyanobacterial strains possessing the *mcy*E gene. The *mcy*E gene region is well-established as a molecular marker for the detection and identification of toxic cyanobacteria [13]. This is a more conserved region than are the other *mcy* regions, and therefore it is a reliable segment of the gene for detection of the potential microcystin producers [32]. This region is also necessary for microcystin production [33]. The *mcy*E gene region has been used for the detection of potential microcystin-producers in different environmental samples [19,31,34,35,36,37], and even from lake sediments [38]. Our results showed that microcystin was produced by cyanobacteria, which established only a low biomass in Lake Stechlin (*Microcystis* and *Planktothrix*).

The dominant cyanobacterial taxa (*Dolichospermum* and *Aphanizomenon*) did not show any potential to produce toxins. Toxin-producing genes corresponding to those taxa were also not detected in the field samples. The toxin analysis results were supported by earlier findings, in which no clear correlation were found between the composition and density of a specific bloom and the concentration of toxins in cyanobacterial cells or in the surrounding water, since not all species or strains are toxic [39]. Several variants of microcystin (LR, LL, and LW) were detected in the field samples from Lake Stechlin. Fastner *et al.* [40] detected different variants of microcystin produced *by Microcystis* sp. and *Planktothrix* sp. in water bodies of Germany. Moreover, strains of *Dolichospermum planktonica* and *Aphanizomenon flos-aquae* have been isolated from Lake Stechlin possessing the saxitoxin-producing gene [18].

The occurrence of a microcystin producing genotype of *P*. *rubescens* has been reported already in Lake Stechlin [19]. In this study, the PCR-amplification of the *mcy*E gene was successfully achieved only in one of two strains of *P*. *rubescens* (P1). This result, based on a molecular approach, was evidenced by the toxin analysis, as the [Dha^7^]MCYST-RR variant was detected from this isolated strain. We also screened isolated strains of *Dolichospermum circinale* (two strains), *Aphanizomenon flos-aquae* (one strain), *Microcystis* cf. *aeruginosa* (two strains) but did not detect any gene responsible for cyanotoxin production (microcystin, saxitoxin or anatoxin-a). Nevertheless, the presence of *Microcystis*, with the potential to produce microcystin in Lake Stechlin, could be shown here for the first time by obtaining a sequence of the *mcy*E fragment from a field sample. Analytical results of field samples also evidenced the presence of microcystins in Lake Stechlin.

During the incidence of cyanobacterial mass development in July 2011, *P*. *agardhii* was present in Lake Stechlin along with the dominating *Dolichospermum circinale*. We could get only sequences of the *mcy*E gene related to *Microcystis* from screened clones of the studied field sample. Toxicity is not species-specific in cyanobacteria, and toxin production varies greatly among the different strains of the same species [41]. Studies have shown that the ability to produce toxins can vary temporally and spatially at a particular site, or within the bloom itself [42]. To find strains with the potential of microcystin production, it is necessary to isolate a number of strains of *Microcystis* and *Planktothrix*. Molecular detection of potential toxin-producing cyanobacterial candidates is a fast prognostic technique for determining the presence or absence of cyanotoxins in a habitat, especially as it is an advantageous method of screening samples before submitting them to quantitative toxicological analyses, thus to reduce monitoring of expenditure.

A higher microcystin amount (27.32 µg L^−1^ LR equivalent) was detected in July 2011, as compared to the sample from November 2012 (*i*.*e*., 4.25 µg L^−1^ LR equivalent). One reason could be the more suitable temperature (20.6 °C) in July 2011 in contrast to November 2012 (9.3 °C). The optimum temperature for toxin production in cyanobacteria is between 20 and 25 °C [43]. The two main factors that have been shown to affect toxin production are light and temperature [43,44], which suggests that cyanobacteria are most toxic during bright periods with warm weather, as is typical for summer in NE Germany. Besides light and temperature, nutrients such as nitrogen [45,46], phosphorus [46] or iron [47] might be important factors that influence the production of cyanotoxins. However, in the nutrient-poor lake studied, epilimnetic concentrations of N and P did not change over the last decades.

The microscopic identification of cyanobacterial taxa that thrived in Lake Stechlin in 2011 and 2012 is supported by evidence from molecular markers. The V3 and V4 are hypervariable regions of 16S rRNA gene and are considered to be the most suitable to discriminate bacterial taxa, including cyanobacteria [48,49]. The present study on the 16S rRNA gene (V3 and V4 regions) is an addition to the phylogeny of *Microcystis*, as sequences of these regions have not been available in GenBank. In our study, the 16S rRNA gene phylogeny of *Microcystis*, based on the variable regions V3–V4, showed a topology of the tree that is congruent to earlier phylogenetic trees with long sequences as different species of *Microcystis* formed a tight cluster [50,51]. The clustering of the Lake Stechlin phylotypes with strains from other continents showed that *Microcystis* sp. strains seem to have no geographic restriction. The high overall diversity and wide global distribution with the lack of phytogeographic structures suggest that *Microcystis* spp. might have a truly cosmopolitan distribution [52]. Our study also provides information on the phylogeny of the genus *Planktothrix* and *Dolichospermum* with the V3 and V4 regions of 16S rRNA gene obtained from Lake Stechlin. *Aphanizomenon* and *Dolichospermum* phylotypes are represented by distinct clades comprised of sequences from different parts of the world. Nevertheless, the *Aphanizomenon* phylotype of Lake Stechlin showed genetic divergence, as it did not cluster with sequences from Germany.

## 4. Experimental Section

### 4.1. Site Description and Sampling

The main morphological and limnological parameters, as well as the results of experiments, were summarised by Casper [1], Casper and Koschel [53], and Koschel and Adams [54]. The surface area is 4.25 km^2^, the maximal depth is 69.5 m, the mean depth is 22.8 m and the total volume of the lake is 98.7 × 10^6^ m^3^. The catchment area is 12.4 km^2^, of which 96% is covered by forest. A bathymetric map of the lake with the sampling locations is shown in Figure 1.

Pelagic phytoplankton samples from the deepest point of the lake were collected biweekly during stratification and monthly during mixed regimes. Additionally, during mass developments of cyanobacteria with scum-development at the shoreline, scum samples were collected in July 2011 and November 2012, pooled and filtered onto glass fibre filters (GF/C, Whatman, Maidstone, UK). Physical parameters (temperature, conductivity, pH, oxygen concentration, oxygen saturation and Secchi transparency) were measured and concentrations of total and dissolved phosphorous and nitrogen (TP, TN, SRP and DIN) were analysed according to Padisák *et al.* [23].

### 4.2. Biomass Calculation, Culturing and Microscopy

The phytoplankton was counted according to Utermöhl’s procedures [55] in sedimentation chambers (Hydro-Bios Apparatebau GmbH, Kiel, Germany) under an inverted microscope Zeiss Axiovert (Zeiss, Oberkochen, Germany). Phytoplankton biomass was calculated by geometric approximations using the computerised counting programme OPTICOUNT version 8 (SequentiX, Klein Raden, Germany, 2012) [56]. The clonal culture of the strains of *Dolichospermum* (A1, A5 and A7), *Aphanizomenon* (AP1), *Planktothrix* (P1, P2) and *Microcystis* (M1, M2) were established by isolating filaments and cells using a micropipettes method. The strains were maintained at the algal culture collection of the Leibniz-Institute of Freshwater Ecology and Inland Fisheries in a suspension using a Z8 medium [57] for *Microcystis* and *Dolichospermum* and a modified Bourrelly medium [58] for *Aphanizomenon* and *Planktothrix*. The cultures were stored at 20 °C and under a 14 h:10 h light-dark cycle. Strains were identified based on morphological traits according to Komarková-Legnerová and Eloranta [59], Komárek and Anagnostidis [60], Komárek and Anagnostidis [61], and Komárek and Komárková [62]. We consequently used the revised designations of genera according to the latest studies: *Planktothrix* (syn. planktonic *Oscillatoria*, Castenholz [63]; Suda *et al.* [64]; Komárek and Komárková [65]), *Dolichospermum* (syn. planktonic *Anabaena*, Wacklin *et al.* [66]).

### 4.3. Molecular Analyses

The genomic DNA was extracted from pooled phytoplankton field samples from July 2011 and November 2012, as well as from cyanobacterial strains of the IGB algal collection using the Dynabead DNA Direct System I (Invitrogen/Dynal Biotech, Oslo, Norway), following the steps outlined in the manufacturer’s manual. The polymerase chain reactions (PCR) were performed in a Peltier Thermal Cycler PTC 200 (MJ Research Inc., San Francisco, CA, USA). The volume and concentrations of the PCR cocktail used were as described by Dadheech *et al.* [67]. Primers CYA361f and CYA785r [48] were employed for the amplification of the V3–V4 regions of the 16S rRNA gene. Amplification of the 16S rRNA gene fragment was carried out as follows: initial three min at 94 °C, 30 cycles of one min at 94 °C, 30 s at 55 °C, 45 s at 70 °C, and a final elongation step at 72 °C for five min. Amplification of the aminotransferase (AMT) domain situated on the modules *mcy*E and *nda*F of the microcystin and nodularin synthetase enzyme complexes was performed using HEPF and HEPR primers [30] with a PCR protocol described earlier [33]. Besides testing the *mcy*E gene for microcystin in the samples, other toxin-producing genes for saxitoxin and anatoxin were examined using suitable primers that had been utilized by earlier workers [18,68,69,70]. The PCR cycling protocol for each primer pair was applied as previously described by the authors.

The amplified products were cleaned using QIAquick PCR purification columns according to the manufacturer’s protocol and were examined on a 1% agarose gel. Cleaned PCR products of field samples were cloned using the Zero Blunt^®^ Topo^®^ PCR cloning kit (Invitrogen, Darmstadt, Germany) according to the manufacturer’s instructions. The positive clones selected were PCR amplified and then cycle sequenced to retrieve the sequence of the AMT domain for the *mcy*E gene. Uncultured *Planktothrix* sp. clones that were sequenced were designated as ST_mcyE_plank. Likewise, the uncultured *Microcystis* sp. clones that were sequenced were entitled ST_mcyE_micro. Both strands of purified products were sequenced on an ABI 3100 Avant Genetic Analyzer using BigDye Terminator Cycle Sequencing Kit v3.1 (Applied Biosystems, Applera Deutschland GmbH, Darmstadt, Germany), as described in the manufacturer’s manual.

The sequences of the 16S rRNA gene belonging to *Dolichospermum*, *Aphanizomenon*, *Planktothrix* and *Microcystis* taxa, and for the AMT domain related to the *Microcystis* and *Planktothrix* taxa, were retrieved from the nucleotide NCBI database and aligned with sequences obtained in the present study using the MUSCLE software [71]. Alignment was visually checked using the Manual Sequence Alignment Editor, Align v05/2008 (SequentiX, Klein Raden, Germany, 2001) [72]. The phylogenetic trees were constructed by the maximum likelihood (ML) method using the programme MEGA version 6.0 [73] with the default settings, applying a suitable model of nucleotide substitution. Confidence values for the edges of the maximum-likelihood tree were computed by bootstrapping of 1000 replications. The nucleotide sequences reported in this study were deposited in the NCBI database under the GenBank accession numbers KM376423–KM376430 (16S rRNA gene), and KM376431–KM376433 (*mcy*E gene).

### 4.4. Toxin Analyses

#### 4.4.1. Quantification of the MCYSTs

For analysis, cells that were collected on glass fibre filters from a known volume were frozen and stored at −20 °C. Prior to freezing, the samples were divided into two parts; the first part (for analysis of cyanotoxins) was frozen (−20 °C) and thawed twice and then filtered through a microfibre filter (GF/C, Whatman), while the second part was lyophilised for determination of dry mass. Microcystin variants were analysed in the whole extracts of the samples. The toxin concentration of the extracts was determined by micellar electrokinetic chromatography, as published earlier [74,75] (MEKC) (Prince CEC-770 instrument; polyimide coated fused silica capillary (Supelco, 60 cm, 50 µm id., effective length: 52 cm); hydrodynamic injection 100 m bar 9 s^−1^; applied voltage: +25 kV; 25 mM sodium-tetraborate-100 mM SDS buffer, pH: 9.3; detection by diode-array detector at 239 nm). DAx 3D version 8.1 software (PrinCE Technologies; Emmen; The Netherlands) was used for the evaluation of the electropherograms. MC-LR was used as a standard (purified in our laboratory, purity approx. 97% (HPLC-DAD)).

#### 4.4.2. MALDI-TOF MS Analysis

Lyophilised waterbloom-samples were screened for microcystins by MALDI-TOF MS. For the analyses, five mg of the lyophilised sample was mixed with 200 μL of 50% aqueous methanol, sonicated for five min, and allowed to stand for one h. The samples were examined in the positive-ion mode using a Bruker Biﬂex MALDI-TOF mass spectrometer equipped with delayed-ion extraction. A 337-nm nitrogen laser was used for desorption/ionisation of the sample molecules. Spectra from multiple (at least 100) laser shots were summarised using a 19-kV accelerating and 20-kV reﬂectron voltage. External calibration was applied using the [M−Na^+^] 1 peaks of malto-oligosaccharides dp 3–7, *m/z* values 527.15, 689.21, 851.26, 1013.31, and 1175.36, respectively. The measurement was performed in the 2,5-dihydroxybenzoic acid (DHB) matrix by mixing 0.5 µL of the matrix solution with 0.5 µL of the sample on the sample target and allowing it to dry at room temperature. The DHB matrix solution was prepared by dissolving DHB (10 mg) in a mixture (0.5 mL) of ethanol and water (1:1, *v*/*v*). The compounds were identiﬁed on the basis of the mass of [M + H]^+^ peak. After the determination of mass values, post-source decay (PSD) measurements were performed directly from the same sample on the template, and microcystins and other peptides were identified by the PSD fragment structure analysis [76].

## 5. Conclusions

In the oligo- to mesotrophic Lake Stechlin, cyanobacteria mass developments in 2011 and 2012 were dominated by the *Aphanizomenon* and *Dolichospermum* taxa. The toxicological analysis revealed different MCYST variants in the field samples. The molecular biological analysis of the *mcy*E gene fragments did show that *Microcystis aeruginosa* and *Planktothrix rubescens*, both minor members of the community, were the microcystin producers in Lake Stechlin. The present study clearly showed that, while focusing on the main players (bloom forming) in lake ecosystems, it is essential to study other cyanobacterial taxa present in the minority. Those should be taken into consideration during the monitoring of toxic cyanobacterial blooms (e.g., in drinking and recreational water sources). This is the first report of the *mcy*E gene fragment of microcystin genes amplified from field samples, and of the simultaneous detection of toxins in the oligo–mesotrophic Lake Stechlin. All lakes of this type were endangered by mass developments of cyanobacteria with potential toxin production due to global or local changes. The findings of this study contribute to the understanding of toxin presence in nutrient-poor lakes.
